# Effect of Printing Orientation on the Dimensional Accuracy of 3D-Printed Denture Base

**DOI:** 10.3390/jfb17030109

**Published:** 2026-02-24

**Authors:** Ivet Dzhondrova, Ilia Liondev, Todor Bogdanov, Todor Uzunov, Nickolay Apostolov, Rangel Todorov, Dimitar Kirov

**Affiliations:** 1 Department of Prosthetic Dental Medicine, Faculty of Dental Medicine, Medical University of Sofia, 1000 Sofia, Bulgaria; i.lyondev@fdm.mu-sofia.bg (I.L.); uzunov@fdm.mu-sofia.bg (T.U.); n.apostolov@fdm.mu-sofia.bg (N.A.); r.todorov@fdm.mu-sofia.bg (R.T.); d.kirov@fdm.mu-sofia.bg (D.K.); 2 Department of Physics and Biophysics, Medical University of Sofia, 1000 Sofia, Bulgaria; tbogdanov@medfac.mu-sofia.bg

**Keywords:** complete denture, additive manufacturing, CAD/CAM, photopolymer resin, build angle, dimensional accuracy

## Abstract

Additive manufacturing is now an integral part of digital prosthodontic workflows, and although stereolithography (SLA) is widely used for denture base fabrication, the dimensional accuracy of printed dentures remains highly dependent on manufacturing parameters, particularly build orientation. This study evaluated the influence of build orientation on the trueness and precision of SLA-printed maxillary and mandibular denture bases. Thirty complete denture bases were fabricated using SLA and divided into three groups according to build orientation: 0°, 45°, and 90° (*n* = 10). The intaglio surfaces of the printed dentures were scanned and compared with their corresponding digital reference models using three-dimensional inspection software. Trueness was quantified using root mean square error (*RMSE*) and directional deviations, while precision was assessed based on the variability of *RMSE* values within each group. Statistical analysis was performed using one-way ANOVA and Tukey’s post hoc test (*p* ≤ 0.05). Build orientation significantly affected the trueness of maxillary denture bases, with dentures printed at 90° demonstrating the lowest *RMSE* values. No statistically significant differences in trueness were observed among build orientations for mandibular denture bases. Precision was not influenced by build orientation for maxillary dentures, whereas mandibular dentures printed at 90° exhibited significantly greater variability compared with 0° and 45°. Build orientation is a critical factor influencing the dimensional accuracy of SLA-printed denture bases in an arch-dependent manner. Optimizing build orientation may enhance both accuracy and reproducibility, thereby improving the predictability and clinical reliability of additively manufactured denture bases.

## 1. Introduction

Additive manufacturing has rapidly evolved from an experimental technology into an established component of contemporary dental laboratory workflows. Within removable prosthodontics, three-dimensional (3D) printing offers several advantages over conventional fabrication techniques, including reduced production time, lower material consumption, and the ability to accurately reproduce complex geometries with minimal manual intervention [1,2,3]. Among the available additive manufacturing technologies, stereolithography (SLA) has gained particular prominence for denture base fabrication due to its high dimensional resolution, reliable reproduction of fine surface details, and predictable photopolymerization behavior. These characteristics have positioned SLA-printed denture bases as a viable alternative to traditionally processed polymethyl methacrylate (PMMA) dentures within fully digital prosthodontic workflows [4,5].

Despite these advantages, the quality and clinical performance of 3D-printed denture bases remain highly dependent on multiple variables throughout the manufacturing process. Altarazi et al. categorized these variables into two major groups: non-controllable factors, such as resin composition, light source characteristics, and polymerization wavelength, and controllable factors, including printing orientation, post-curing temperature, and polymerization time [6]. Prior studies have consistently demonstrated that, due to the multistep nature of additive manufacturing of denture bases, deviations introduced at any stage may significantly affect dimensional accuracy, surface characteristics, mechanical behavior, and adaptation to the supporting tissues [7,8,9,10].

Among the controllable parameters, build orientation is consistently identified as one of the most influential factors affecting the accuracy of printed objects. The selected orientation determines the direction of layer stacking, the distribution and number of support structures, the location of slicing planes, and the degree of light exposure during polymerization. Consequently, build orientation influences not only surface quality and mechanical performance but also the spatial accuracy of the printed denture base, often described in terms of trueness and precision according to ISO standards [11,12,13].

According to ISO 5725-1, accuracy encompasses both trueness, which reflects the agreement between a manufactured object and its reference geometry, and precision, which describes the consistency among multiple objects produced under identical conditions [14]. In addition, long-term dimensional stability remains a critical requirement for denture bases intended for prolonged service in the oral environment, as dimensional changes over time may adversely affect fit, retention, and patient comfort. In the context of additive manufacturing, evaluating both trueness and precision provides a more comprehensive assessment of the reliability of a given printing strategy. While trueness reflects the degree of agreement between a printed denture base and its digital reference design, precision describes the consistency and reproducibility of repeated prints produced under identical conditions. Consequently, a denture base may exhibit acceptable trueness yet demonstrate poor reproducibility across repeated manufacturing cycles, which may compromise clinical predictability and standardization of digital workflows [15,16].

Several studies have emphasized the importance of selecting an appropriate build orientation to achieve clinically acceptable dimensional accuracy in additively manufactured dental restorations. Shim et al. reported that printing orientation plays a decisive role in achieving high dimensional fidelity of SLA-printed dental objects, directly influencing surface accuracy and overall geometric stability [17]. Unkovskiy et al. [8] demonstrated that a 45° build orientation resulted in superior trueness compared with both horizontal (0°) and vertical (90°) orientations, a finding further corroborated by Song [11]. Despite its accuracy advantages, a 45° orientation is associated with notable practical trade-offs, including increased support structure requirements, higher material consumption, and extended post-processing time. In contrast, vertical printing at 90° typically reduces resin usage due to more efficient support placement but substantially increases build time as a result of greater object height. Horizontal printing at 0° offers the shortest production time; however, it is often accompanied by reduced dimensional accuracy, particularly in flat or extensive surface regions. These findings underscore the necessity of balancing accuracy, efficiency, and material consumption when selecting build orientation for denture base fabrication.

Other investigations have focused on the influence of build orientation on the adaptation of denture bases to the supporting soft tissues. Jin et al. reported no statistically significant differences in adaptation between orientations but nevertheless recommended printing maxillary denture bases at 135° and mandibular bases at 100° to optimize clinical fit [18]. Yoshidome et al. similarly observed that denture bases printed at 45° and 225° demonstrated the most favorable adaptation to the denture-bearing area, with accuracy comparable to or slightly exceeding that of conventionally fabricated dentures [19].

Further evidence was provided by Cameron et al. [20] and Hada et al. [13], who reported improved trueness and adaptation when denture bases were printed at 45° or 90°, particularly when additional internal support structures were employed. In contrast, Yan et al. observed superior accuracy for horizontally printed denture bases and the greatest deviations at 90°, while also noting that maxillary dentures generally exhibit fewer volumetric distortions than mandibular dentures [16]. Kim et al. presented yet another perspective, identifying the vertical 90° orientation as the most accurate and the horizontal 0° orientation as the least precise [21]. Taken together, these findings indicate that the influence of build orientation on denture base accuracy remains complex and system-dependent.

Given the increasing reliance on fully digital workflows in prosthetic dentistry, clarifying the effects of build orientation on both trueness and precision is essential for optimizing manufacturing strategies and improving clinical outcomes. Therefore, the aim of this study was to evaluate the influence of build orientation (0°, 45°, and 90°) on the dimensional accuracy (trueness and precision) of SLA-printed denture bases.

## 2. Materials and Methods

### 2.1. Study Design

A total of 30 complete denture bases were fabricated using additive manufacturing, including 15 maxillary and 15 mandibular dentures. All specimens were divided into three experimental groups according to build orientation during the printing process:

- Group A (*n* = 10): 0° orientation
- Group B (*n* = 10): 45° orientation
- Group C (*n* = 10): 90° orientation

The sample size was based on previously published in vitro studies evaluating the dimensional accuracy of additively manufactured denture bases, in which group sizes typically range between 6 and 10 specimens per build orientation [13,16,20,21].

In the present study, each build orientation group included 10 denture bases in total (*n* = 5 maxillary and *n* = 5 mandibular). The maxillary and mandibular dentures were analyzed separately to allow arch-specific evaluation.

Therefore, the sample size per arch (*n* = 5) in the present study was smaller than that reported in some previous investigations. No formal a priori sample size calculation was performed, which should be considered when interpreting the findings.

The study was based on a clinical case of complete edentulism. An initial alginate impression was obtained and used to fabricate a custom impression tray, followed by a functional impression procedure to capture the definitive denture-bearing anatomy. The resulting working casts were digitized using a laboratory scanner (F8 Lab Scanner, 3Shape, Copenhagen, Denmark) to generate high-resolution digital models.

### 2.2. Digital Denture Base Design, Fabrication, and Post-Processing

**Figure 1:**
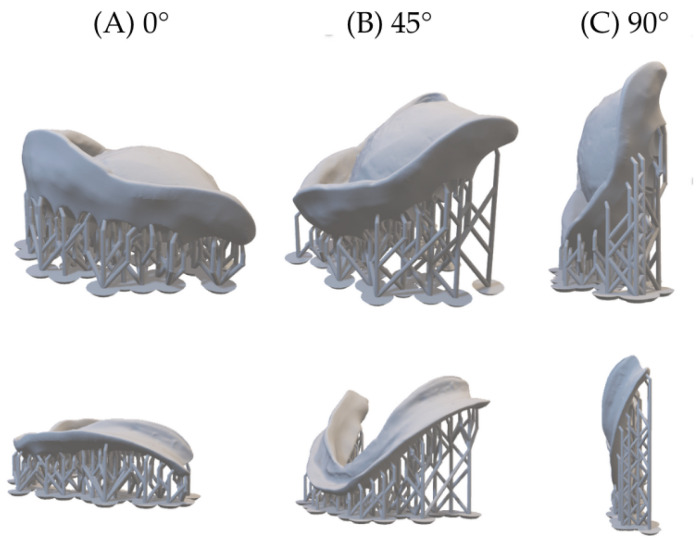
Computer-aided design of denture bases (**A**) 0° orientation (horizontal): Denture base printed flat on the build platform. (**B**) 45° orientation (angled): Denture base printed at a 45° tilt to modify layer deposition and support placement. (**C**) 90° orientation (vertical): Denture base printed upright, with layers deposited along the height.

Denture bases were designed using dental CAD software (DentalCAD 3.0 Galway; exocad GmbH, Darmstadt, Germany) according to standard prosthodontic design principles. The finalized designs were exported as STL files (Figure 1) and imported into Phrozen DS Slicer V4.3.2 (Phrozen Tech Co., Ltd., Hsinchu, Taiwan) for print preparation. All denture bases were fabricated using a masked stereolithography (MSLA) 3D printer (Sonic XL 4K Plus; Phrozen Tech Co., Ltd., Hsinchu, Taiwan) in combination with a denture base photopolymer resin (NextDent Denture 3D+; NextDent, 3D Systems, Soesterberg, The Netherlands). The printer operates at a native resolution of 3840 × 2400 pixels, providing high spatial accuracy in the X–Y plane. A layer thickness of 50 μm was selected for all specimens to balance dimensional accuracy and printing efficiency.

Printing parameters were standardized across all groups. The base layers were printed using six initial layers with an exposure time of 40 s per layer, followed by normal layers with an exposure time of 4 s. A lift height of 8 mm was used for base layers and 7 mm for normal layers, with peel and return speeds set to 50 mm/min and 100 mm/min, respectively. Build orientation was the only variable altered between experimental groups; all other printing conditions were kept constant in accordance with the manufacturer’s recommendations.

Following printing, all dentures underwent a standardized post-processing protocol. Specimens were first washed in 95% isopropyl alcohol using an ultrasonic bath (Phrozen Tech Co., Ltd., Hsinchu, Taiwan) to remove uncured resin. Subsequently, post-polymerization was performed in a Phrozen Cure unit (Phrozen Tech Co., Ltd., Hsinchu, Taiwan) for 30 min at 80 °C under 405 nm ultraviolet light, ensuring complete polymerization and stabilization of the material properties. Support structures were not removed prior to scanning in order to avoid unintended alteration of the denture base geometry. All supports were located on the external surface, and the intaglio surface used for trueness and precision analysis remained unaffected.

After post-curing, the intaglio surface of each printed denture base was scanned using the same calibrated laboratory scanner (F8 Lab Scanner, 3Shape, Copenhagen, Denmark; manufacturer-reported accuracy up to 5 μm). Scanning was performed under standardized ambient conditions following the manufacturer’s recommendations. During digitization, each denture base was consistently positioned on the scanner platform with the intaglio surface oriented upward to standardize data acquisition. The same positioning protocol was applied to all specimens to minimize inter-scan variability. No external mounting jig was used; however, the laboratory scanner utilizes automated multi-axis image acquisition and internal alignment algorithms that reduce operator-dependent positioning errors and improve scan reproducibility.

These scans served as the measured models for subsequent geometric analysis. All scans were conducted by a single experienced operator under identical conditions to minimize operator-related variability. The scanned STL files were visually inspected for defects prior to deviation analysis, and only validated datasets were included.

**Figure 2:**
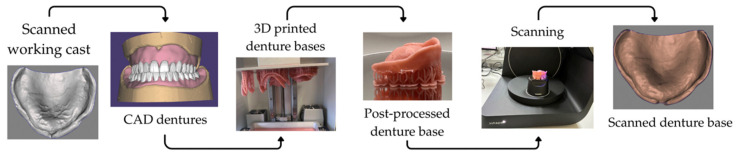
Workflow of denture base fabrication and digitization, including scanning of the working cast, CAD denture design, 3D printing, post-processing, and scanning of the printed denture base.

A schematic overview of the experimental workflow is presented in Figure 2.

### 2.3. Trueness and Precision Analysis

The evaluation of dimensional accuracy was performed in accordance with ISO 5725-1:1994 [14], which defines the concepts of trueness and precision for measurement methods. Although no specific ISO standard was applied to the digital scanning procedure itself, the scanning and CAD-to-scan comparison workflow followed protocols commonly used in digital prosthodontic research.

**Figure 3:**
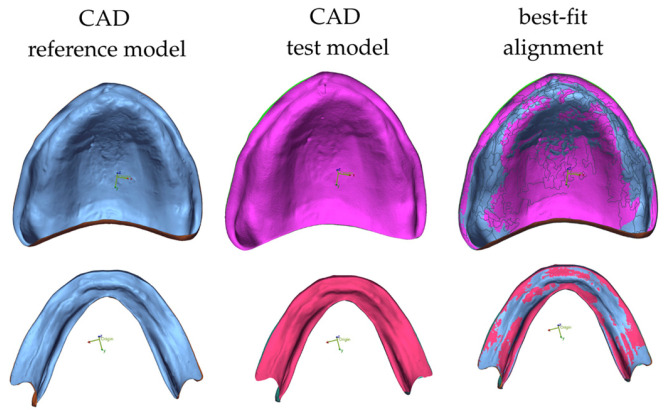
Three-dimensional alignment workflow showing the CAD reference model, CAD test model, and their superimposition following best-fit alignment for trueness analysis.

Geometric evaluation was conducted using Geomagic Control X 64 (3D Systems, Rock Hill, SC, USA). The original CAD denture base design was designated as the CAD reference model (CRM), while the scanned intaglio surface of each printed denture base was defined as the CAD test model (CTM). The datasets were aligned using a best-fit alignment algorithm, ensuring maximal surface correspondence between the reference and measured models (Figure 3).

For deviation analysis, the nominal tolerance range was set to ±50 μm, which has been widely reported in the literature as a clinically acceptable threshold for denture base adaptation, while the maximum comparison range was set to ±500 μm [16,21,22]. Dimensional accuracy was quantified by calculating the root mean square error (*RMSE*) using the following equation:

$$RMSE=\frac{1}{\sqrt{n}}\cdot \sqrt{\sum_{i=1}^{n}{\left(X_{1,i}-X_{2,i}\right)}^{2}}$$

where

- *X*_1,*i*_ represents the coordinate of point *i* on the reference model,
- *X*_2,*i*_ represents the coordinate of point *i* on the measured model,
- *n* is the total number of measured points.

In the present analysis, trueness was quantified using the root mean square error (*RMSE*), defined as the deviation between the printed denture bases and their corresponding digital reference models. Precision was evaluated by assessing the reproducibility of the printing process, expressed as the variability of *RMSE* values within each build orientation group. In addition to *RMSE*, mean positive deviation and mean negative deviation values were calculated to characterize volumetric discrepancies between the printed denture bases and their digital designs.

For precision assessment, the scanned intaglio surfaces of the printed denture bases within each build orientation group were compared pairwise. For each group (*n* = 5), all possible unique scan-to-scan combinations were generated, resulting in 10 pairwise comparisons per build orientation. These datasets were aligned using the same best-fit alignment algorithm applied for trueness evaluation, but without reference to the original CAD model. Precision was quantified by calculating the *RMSE* for each pairwise comparison, thereby reflecting the variability among repeated prints produced under identical manufacturing conditions.

For visual assessment, colorimetric deviation maps were generated for each comparison. The deviation scale was divided into three clinically relevant zones:

−0.05 to +0.05 mm: optimal adaptation (green)
- +0.05 to +0.5 mm: increased volume relative to the design (warm colors)
−0.05 to −0.5 mm: decreased volume relative to the design (cold colors)

### 2.4. Statistical Analysis

Statistical analysis was performed using IBM SPSS Statistics for Windows, Version 22.0 (IBM Corp., Armonk, NY, USA). The normality of data distribution was assessed using the Shapiro–Wilk test, while homogeneity of variances was verified using Levene’s test. Mean *RMSE* values, mean positive deviations, and mean negative deviations among the three build orientation groups (0°, 45°, and 90°) were compared using one-way analysis of variance (ANOVA). When statistically significant differences were identified, Tukey’s post hoc test was applied for pairwise comparisons. A significance level of *p* ≤ 0.05 was set. A post hoc power analysis (α = 0.05) based on the observed effect sizes was performed to evaluate the achieved statistical power for both trueness and precision outcomes (Supplementary Table S1).

## 3. Results

The accuracy results for the maxillary denture bases printed at 0° (Group A), 45° (Group B), and 90° (Group C) are summarized in Table 1. The *RMSE* values, representing the overall deviation between the measured model (printed denture base) and the reference model (digital design), differed significantly among the three build orientations (*p* = 0.013, η^2^ = 0.51). Dentures printed at 0° showed the highest *RMSE* (0.1445 ± 0.007 mm), indicating the greatest dimensional deviation. In contrast, dentures printed at 90° demonstrated the lowest *RMSE* (0.1195 ± 0.005 mm), corresponding to the highest trueness.

A statistically significant difference (*p* = 0.02) was also observed in the mean positive deviation, which reflects localized convex areas or material excess. The 45° orientation produced the highest mean positive deviation (0.0747 ± 0.005 mm), whereas the 90° orientation exhibited the lowest (0.0655 ± 0.004 mm). In contrast, mean negative deviation, indicating localized concavities or underdeveloped contours, did not differ significantly among the three build-angle groups.

**Figure 4:**
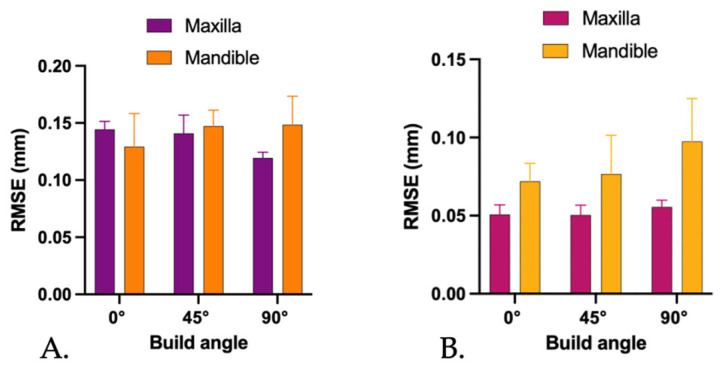
Root mean square error (*RMSE*) values of maxillary and mandibular denture bases fabricated at different build orientations (0°, 45°, and 90°): (**A**) trueness *RMSE* determined by comparison with the CAD reference model; (**B**) precision *RMSE* determined by pairwise comparison of scanned denture bases within each build-orientation group. Error bars represent standard deviation.

The results for the mandibular denture bases fabricated at 0° (Group A), 45° (Group B), and 90° (Group C) are shown in Table 2. Unlike the maxillary dentures, the *RMSE* values for mandibular bases did not differ significantly among the three orientation groups, indicating that build angle had no statistically significant effect on overall trueness (η^2^ = 0.14). Figure 4 presents the comparison of *RMSE* values between maxillary and mandibular denture bases printed at 0°, 45°, and 90° orientations.

Similarly, no statistically significant differences were observed among the groups for mean positive deviation or mean negative deviation. Both convex and concave surface deviations remained comparable across orientations.

Precision analysis revealed different trends for maxillary and mandibular denture bases. For the maxillary dentures, no statistically significant differences in precision were observed among the three build orientations, indicating comparable reproducibility of the printing process regardless of orientation. In contrast, mandibular denture bases demonstrated a significant effect of build orientation on precision (*p* = 0.04). Dentures printed at 90° showed significantly greater variability compared with those printed at 0° and 45°, which exhibited similar and more consistent precision values.

Post hoc power analysis demonstrated adequate power for detecting differences in maxillary trueness and mandibular precision, whereas lower power was observed for the remaining comparisons.

**Figure 5:**
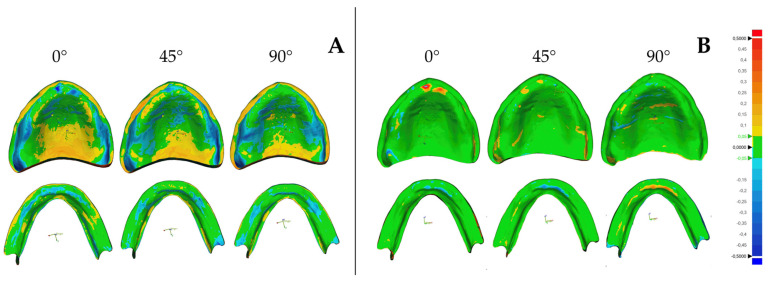
Color-coded deviation maps illustrating dimensional accuracy of denture bases fabricated at different build orientations (0°, 45°, and 90°). (**A**) Trueness maps obtained by comparing the printed denture bases (CAD test models) with their corresponding digital designs (CAD reference models). (**B**) Precision maps obtained by pairwise comparison of scanned denture bases printed under identical conditions within each build-orientation group.

Color deviation maps generated after alignment of the digital reference model with the printed maxillary and mandibular denture bases are presented in Figure 5, illustrating the spatial distribution of surface deviations for trueness and the reproducibility patterns used to assess precision across build orientations.

## 4. Discussion

The present study evaluated the influence of build orientation on the trueness of maxillary and mandibular denture bases fabricated using additive manufacturing. The findings indicate that build angle significantly affected the accuracy of maxillary denture bases, whereas no statistically significant differences were observed among build orientations for mandibular dentures.

In a previous study, the authors reported that build orientation markedly affected the mechanical performance of 3D-printed denture base materials, including flexural strength and hardness [23]. The present study complements these results by demonstrating that the same manufacturing parameter also significantly influences trueness and precision, underscoring the multifactorial impact of build orientation on clinical performance.

Among the maxillary groups, the 90° build orientation demonstrated the lowest *RMSE* values (0.1195 ± 0.005 mm) and the lowest mean positive deviation (0.0655 ± 0.004 mm), indicating superior overall accuracy and fewer localized convex discrepancies on the intaglio surface. Conversely, dentures printed at 0° showed the highest *RMSE* (0.1445 ± 0.007 mm), reflecting greater volumetric distortion and reduced adaptation fidelity.

The 90° orientation exhibited high three-dimensional accuracy, with deviations remaining within the clinically acceptable threshold of ±100 µm, and with the majority of surface deviations remaining within the clinically acceptable threshold of ±50 µm. The ±50 µm tolerance is widely recognized in the literature as a clinically acceptable limit for denture-base adaptation [13,22,24]. From a clinical perspective, the accuracy of the intaglio surface is crucial for achieving optimal tissue adaptation, improved retention, patient comfort, and reduced need for post-delivery adjustments. The higher accuracy observed in the 90° group suggests that vertical printing may represent a reliable and predictable approach for SLA-based denture fabrication within the tested system.

These findings are consistent with the results of Kim et al. [21], who also reported the highest accuracy at 90° and the poorest at 0°. Similar tendencies were noted by Hada et al. [13], who recorded the lowest *RMSE* at 45°, followed by 90° and 0°, and by Cameron et al. [20], who demonstrated improved adaptation at 45° and 90° compared with 0°. Yoshidome et al. [19] likewise found optimal adaptation at 45° and 225°. Unkovskiy et al. [8] and Song [11] also identified 45° as advantageous for achieving high trueness, although they noted increased material consumption and longer print times at this orientation.

In contrast, the results partially diverge from those of Yan et al. [16], who reported superior accuracy at 0° and the largest deviations at 90°. Such discrepancies may arise from differences in printing technology (DLP vs. SLA), exposure parameters, or resin type, as variations in photopolymer material behavior—including polymerization shrinkage and layer stacking—can substantially affect dimensional accuracy.

Some authors suggest [8,20] that vertical printing (90°) results in a more uniform distribution of internal stresses and reduced deformation because layers are stacked vertically rather than across broad horizontal surfaces. This arrangement improves layer detachment from the resin vat and minimizes cumulative *z*-axis errors commonly associated with horizontal printing. From a production perspective, printing at 90° also reduces material consumption due to more efficient support placement. However, vertical printing is typically associated with increased print time due to increased build height. In contrast, a 0° orientation involves depositing layers across larger surface areas, making the print more susceptible to uneven polymerization shrinkage, stepped artifacts, and distortion in flatter anatomical regions such as the posterior palate. Such tendencies have been documented in other photopolymerization-based studies [13,25,26]. The 45° orientation, often considered a compromise, yielded clinically acceptable values in the present study, likely due to uneven internal stress distribution and localized deformations introduced during post-curing.

Notably, build orientation did not significantly influence the accuracy of mandibular denture bases in this study. This finding may be attributed to differences in anatomical geometry and surface complexity: mandibular bases exhibit shallower vestibules and a less complex intaglio morphology, reducing the susceptibility to deformation during polymerization and layer stacking. Similar observations were reported by Jin et al. [18], who also found no statistically significant influence of build orientation on mandibular denture adaptation but recommended specific angles (135° for maxillary and 100° for mandibular bases) to balance accuracy and printing time.

Analysis of the deviation maps revealed that negative deviations predominantly occurred along the crest of the alveolar ridge, whereas positive deviations were concentrated along the peripheral borders. In maxillary dentures, additional positive deviations were consistently observed across the palatal surface and the posterior palatal seal, especially in the 0° group. These patterns may be explained by nonuniform polymerization shrinkage and the accumulation of internal stresses characteristic of photopolymer-based 3D printing. Horizontal layer deposition (0°) over large surface areas promotes stepped artifacts and concavities along the ridge, while vertical stacking (90°) can induce shrinkage along the height of the object. Positive deviations along borders and flatter regions may result from resin pooling, localized material accumulation, or insufficient support density, which allow slight material spreading during polymerization.

Regarding precision, the present study demonstrated an arch-dependent effect of build orientation on the reproducibility of SLA-printed denture bases. For maxillary dentures, precision did not differ significantly among build orientations, indicating comparable reproducibility regardless of printing angle. This finding is consistent with Yan et al. [16], who similarly reported that build orientation did not significantly influence maxillary precision, suggesting that the larger supporting area and more uniform geometry of maxillary denture bases may promote stable and repeatable printing outcomes.

In contrast, mandibular denture bases in the present study exhibited significantly reduced precision when printed at a 90° orientation, with greater *RMSE* variability compared with 0° and 45°. This observation aligns with the findings of Yan et al. [16], who also identified increased variability in mandibular precision at 90°, and supports the notion that mandibular denture morphology, characterized by thinner cross-sections and more complex contours, may be more sensitive to vertical layer stacking.

In another study [13], precision was highest at a 45° orientation and lowest at 0° and 90°, attributing this trend to differences in layer orientation and platform positioning. While the present study did not identify a single optimal orientation for mandibular precision, both investigations confirm that precision values are consistently lower than trueness values, indicating that SLA systems generally exhibit good reproducibility even when absolute dimensional accuracy varies. The predominantly green color distribution observed in the precision deviation maps further supports this finding, reflecting consistent spatial deviation patterns across repeated prints.

This study has several limitations that should be considered when interpreting the findings. First, the investigation was limited to a single SLA printer–resin combination and a specific post-processing protocol; therefore, the results cannot be directly generalized to other printing systems, materials, or curing conditions. Second, although both maxillary and mandibular denture bases were evaluated, the sample size was relatively limited and may not fully represent the full variability encountered in clinical practice; therefore, the findings should be interpreted with appropriate caution. Third, the analysis focused on geometric accuracy under controlled laboratory conditions and did not include artificial ageing, thermal cycling, or functional loading, which may influence dimensional stability over time. Another limitation is the potential influence of optical scanning accuracy, which, despite standardization, may introduce minor measurement uncertainty. Furthermore, the digital scanning procedure was not performed under a dedicated ISO digitization standard, which may limit direct comparison with studies employing fully standardized scanning protocols. Finally, only build orientation was varied, while other potentially influential parameters such as support density, layer thickness, and exposure settings were kept constant. Future studies should incorporate different manufacturing systems, ageing protocols, and multi-factorial designs to further elucidate the long-term clinical performance of additively manufactured denture bases.

Overall, the present findings confirm build orientation as a key determinant of the dimensional accuracy of additively manufactured denture bases. Importantly, the results demonstrate that build orientation influences both trueness and precision in an arch-dependent manner, emphasizing that accuracy cannot be fully characterized by trueness alone. Consideration of both parameters is therefore essential when optimizing printing strategies for clinical denture fabrication. Appropriate selection of build orientation, together with controlled polymerization conditions, is crucial for producing denture bases with predictable accuracy and reliable reproducibility.

## 5. Conclusions

Within the limitations of this study, build orientation significantly influenced the dimensional accuracy of additively manufactured denture bases in an arch-dependent manner. For maxillary dentures, specimens printed at a 90° orientation demonstrated superior trueness compared with 0° and 45° orientations, while precision was not significantly affected. For mandibular denture bases, trueness was not influenced by build orientation; however, precision was significantly lower at the 90° orientation. These findings confirm that build orientation should be considered when optimizing the manufacturing accuracy and reproducibility of 3D-printed denture bases.

## Figures and Tables

**Table 1:** Trueness evaluation of maxillary denture bases printed at different build orientations based on *RMSE* and directional deviation metrics (mean ± SD).

		Trueness			Precision		
	Build Angle	Mean ± SD	F	*p*	Mean ± SD	F	*p*
RMSE	0°	0.1445 ^A^ ± 0.007	6.266	0.013 *	0.0507 ^A^ ± 0.006	2.534	0.098
	45°	0.1409 ^A^ ± 0.016			0.0504 ^A^ ± 0.006		
	90°	0.1195 ^B^ ± 0.005			0.0556 ^A^ ± 0.004		
Mean positive deviation	0°	0.0728 ^A^ ± 0.005	4.806	0.02 *	—	—	
	45°	0.0747 ^A^ ± 0.005					
	90°	0.0655 ^B^ ± 0.004					
Mean negative deviation	0°	−0.123 ^A^ ± 0.006	3.22	0.07	—	—	
	45°	−0.1197 ^A^ ± 0.014					
	90°	−0.1071 ^A^ ± 0.008					

* Statistical significance was set at *p* < 0.05. Cells sharing the same superscript letter are not significantly different from one another.

**Table 2:** Trueness evaluation of mandibular denture bases printed at different build orientations based on *RMSE* and directional deviation metrics (mean ± SD).

		Trueness			Precision		
	Build Angle	Mean ± SD	F	*p*	Mean ± SD	F	*p*
RMSE	0°	0.1292 ^A^ ± 0.029	1.01	0.39	0.072 ^A^ ± 0.011	3.624	0.04 *
	45°	0.1473 ^A^ ± 0.014			0.0767 ^A^ ± 0.024		
	90°	0.1485 ^A^ ± 0.025			0.0974 ^B^ ± 0.027		
Mean positive deviation	0°	0.0537 ^A^ ± 0.005	2.72	0.1	—	—	
	45°	0.0481 ^A^ ± 0.016					
	90°	0.068 ^A^ ± 0.016					
Mean negative deviation	0°	−0.123 ^A^ ± 0.006	3.22	0.07	—	—	
	45°	−0.1197 ^A^ ± 0.014					
	90°	−0.1071 ^A^ ± 0.008					

* Statistical significance was set at *p* < 0.05. Cells sharing the same superscript letter are not significantly different from one another.

## Data Availability

The raw data supporting the conclusions of this article will be made available by the authors on request.

## Supplementary Materials

The following supporting information can be downloaded at: https://www.mdpi.com/article/10.3390/jfb17030109/s1, Table S1: Power analysis.

## Abbreviations

The following abbreviations are used in this manuscript:

CAD	Computer-aided design
CRM	CAD reference model
CTM	CAD test model
RMSE	Root mean square error
SLA	Stereolithography
