# Supplementary material for: Effect of Printing Orientation on the Dimensional Accuracy of 3D-Printed Denture Base
Source: J Funct Biomater. 2026 Feb 24;17(3):109. doi: 10.3390/jfb17030109 (PMC13028307; doi:10.3390/jfb17030109)
Supplement: Supplementary file 1 [file jfb-17-00109-s001.zip › jfb-4160822-supplementary.pdf]

Table S1. Power analysis.

Post-hoc power analysis					
RMSE	Arch	F	η^2	Cohen's f	Power
trueness	Maxillary	6.266	0.51	1.02	0.88
precision	Maxillary	2.534	0.30	0.66	0.39
trueness	Mandibular	1.01	0.14	0.40	0.23
precision	Mandibular	3.624	0.38	0.78	0.62
